# Supplementary material for: End Sequence Analysis Toolkit (ESAT) expands the extractable information from single-cell RNA-seq data
Source: Genome Res. 2016 Oct;26(10):1397–410. doi: 10.1101/gr.207902.116 (PMC5052061; doi:10.1101/gr.207902.116)
Supplement: Supplemental Material [file supp_gr.207902.116_Supp_Materials.tar.gz › ESAT-esat_v0.1/doc/html/classbroad_1_1pda_1_1seq_1_1alignment_1_1_stored_alignment_stats-members.html]

ESAT: Member List


ESAT


- Main Page
- Related Pages
- Packages
- Classes
- Files

- Class List
- Class Index
- Class Hierarchy
- Class Members

All Classes Namespaces Files Functions Variables Enumerator Pages

- broad
- pda
- seq
- alignment
- StoredAlignmentStats

broad.pda.seq.alignment.StoredAlignmentStats Member List

This is the complete list of members for broad.pda.seq.alignment.StoredAlignmentStats, including all inherited members.

|  |  |  |
| --- | --- | --- |
| getAlignmentIdxSizeAtCreation() | broad.pda.seq.alignment.StoredAlignmentStats |  |
| getAlignmentSizeAtCreation() | broad.pda.seq.alignment.StoredAlignmentStats |  |
| getGlobalRPKMConstant() | broad.pda.seq.alignment.StoredAlignmentStats |  |
| getLambda() | broad.pda.seq.alignment.StoredAlignmentStats |  |
| getLastModifiedAlignmentIdxTimeStampAtCreation() | broad.pda.seq.alignment.StoredAlignmentStats |  |
| getLastModifiedAlignmentTimeStampAtCreation() | broad.pda.seq.alignment.StoredAlignmentStats |  |
| getLastModifiedStoredTimeStamp() | broad.pda.seq.alignment.StoredAlignmentStats |  |
| getLocalRpkmConstant() | broad.pda.seq.alignment.StoredAlignmentStats |  |
| getMaskedRegions() | broad.pda.seq.alignment.StoredAlignmentStats |  |
| getNumberOfReads() | broad.pda.seq.alignment.StoredAlignmentStats |  |
| getNumMarkers() | broad.pda.seq.alignment.StoredAlignmentStats |  |
| getReasonForUnsafe() | broad.pda.seq.alignment.StoredAlignmentStats | protected |
| isAlignmentPaired() | broad.pda.seq.alignment.StoredAlignmentStats |  |
| isSafeToLoad() | broad.pda.seq.alignment.StoredAlignmentStats | protected |
| load(File alignmentStoredStatFile) | broad.pda.seq.alignment.StoredAlignmentStats |  |
| SAVED\_MODEL\_STATS\_EXT | broad.pda.seq.alignment.StoredAlignmentStats | static |
| setAsSafeToLoad() | broad.pda.seq.alignment.StoredAlignmentStats | protected |
| setAsUnsafeToLoad() | broad.pda.seq.alignment.StoredAlignmentStats | protected |
| setReasonForUnsafe(String reasonForUnsafe) | broad.pda.seq.alignment.StoredAlignmentStats | protected |
| store(AlignmentDataModelStats alignmentStats) | broad.pda.seq.alignment.StoredAlignmentStats | static |
| StoredAlignmentStats() | broad.pda.seq.alignment.StoredAlignmentStats |  |


---

Generated on Fri Aug 15 2014 15:11:01 for ESAT by  

 1.8.7
